# Improving Imaging Modalities in Early Psoriatic Arthritis: The Role of Ultrasound in Early Diagnosis of Psoriatic Arthritis

**DOI:** 10.3389/fmed.2021.804695

**Published:** 2022-01-07

**Authors:** Tania Gudu, Beverly Ng, Hannah Jethwa, Catherine Graham, Veda Kudva, Jashmitha Rammanohar, Chen Zhang, Mark Sapsford, Deepak R. Jadon

**Affiliations:** ^1^Department of Rheumatology, Cambridge University Hospitals NHS Foundation Trust, Cambridge, United Kingdom; ^2^School of Clinical Medicine, University of Cambridge, Cambridge, United Kingdom; ^3^Queen Mary University, London, United Kingdom; ^4^Department of Rheumatology, Middlemore Hospital, Counties Manukau District Health Board, Auckland, New Zealand; ^5^Department of Medicine, University of Cambridge, Cambridge, United Kingdom

**Keywords:** psoriatic arthritis, ultrasound, early diagnosis, differential diagnosis, psoriasis, enthesitis

## Abstract

**Objective:** Despite recent advances, early diagnosis of psoriatic arthritis (PsA) remains a challenge in clinical practice. Ultrasound (US) could be a useful tool for the diagnosis and management of PsA. The objective of this review was to determine the role of US in early diagnosis of PsA.

**Methods:** We have performed a literature review aiming to evaluate studies on US findings in psoriasis and their predictive value of progression to PsA, as well as studies on US features specific for PsA in comparison with other conditions.

**Results:** A total of 40 studies were included. Sixteen studies assessed US findings in psoriasis, of which only 3 prospectively evaluated the role of US in predicting progression to PsA. Patients with PsA had a greater frequency of US abnormalities, in particular enthesitis and Power Doppler(PD) signal compared to patients with psoriasis only. In the longitudinal studies, psoriatic patients with higher enthesopathy scores at baseline were more likely to progress to PsA. Twenty-four studies evaluated US abnormalities in PsA and compared them to other conditions. Most specific US features that distinguish PsA from psoriasis were PD signal and erosions in joints and entheses. Extra-synovial changes, including peri-tendinous dermal soft tissue oedema with associated PD signal and flexor tendon enthesopathy, as well as thickening of the pulleys in the flexor tendons were highly characteristic for PsA, as they were frequently found in PsA patients, but in none of the RA patients. US-detected entheseal abnormalities in particular erosions and PD signal were more frequent in patients with PsA compared to fibromyalgia.

**Conclusion:** Despite the wide use of US in PsA, more research is needed to identify predictive factors of progression to PsA in patients with psoriasis, as well as to determine most specific US features that differentiate PsA from other conditions.

## Introduction

Psoriatic arthritis (PsA) is estimated to affect between 0.3 and 1% of the population but its prevalence among patients with cutaneous psoriasis varies between 6 and 42% (1). The condition is heterogeneous, with five identified clinical patterns: distal-predominant, oligoarticular asymmetrical, polyarticular, spondylitis and arthritis mutilans (1) and patients can present with synovitis, tenosynovitis, dactylitis, enthesitis, or with a combination of these. The heterogeneity of symptoms, in addition to the lack of serum diagnostic biomarkers as opposed to rheumatoid arthritis (RA), may contribute to a possible diagnostic delay and many patients with PsA can remain undiagnosed for many months or even years (2, 3). Such delays in diagnosis and treatment initiation can result in progression of clinical and radiological damage, resulting in poorer long-term disease outcomes but also impact dramatically on physical function and quality of life (4).

Clinical assessment has its limitations, with variability between healthcare professionals in the identification of subtle inflammatory features (5). As such, the use of imaging to identify pathology in such patients has increased and, in recent years, many research groups have focused on evaluating the sensitivity of imaging modalities to aid with the diagnosis of early PsA (6). Plain film radiography is often easily accessible, low-cost and time-efficient however lacks sensitivity in the identification of early disease; ultrasonography and magnetic resonance imaging (MRI), however, have the advantage of assessing extra-articular structures and are more sensitive at characterizing active inflammation (6). Compared to US, MRI has the advantage of access to sites beyond the acoustic window, as well as identifying bone marrow oedema which is extremely helpful in assessing joints and entheses. However, US is often more routinely available, cheaper and lacks contraindications compared to MRI (6). Furthermore, as a “real-time” imaging modality it allows the clinician to be able to focus on symptomatic sites which may improve sensitivity and the ability to use gray-scale assessment in addition to Power Doppler (PD) provides added benefits (7).

The use of ultrasonography has demonstrated increased sensitivity at detecting synovial hypertrophy and synovitis compared to clinical examination alone (7); as such, it is a useful modality to assess for subclinical inflammatory features. Furthermore, it can aid with differentiating between other causes of arthritis, such as RA, osteoarthritis (OA) or crystal arthropathies (8).

## Ultrasound Findings in Patients With Psoriasis and Predictive Value of Progression to Psoriatic Arthritis

Ultrasound (US) has been proposed to be a valuable tool to assess features of PsA, including enthesitis, joint synovitis, tenosynovitis and dactylitis. A total of 13 studies were identified in this review that evaluated US findings cross-sectionally in patients with psoriasis as summarized in Supplementary Table 1. Three studies performed follow-up to determine the role of early US in asymptomatic psoriatic patients in predicting progression to PsA, as summarized in Supplementary Table 1.

The majority of cross-sectional studies highlighted a higher frequency of US abnormalities in patients with psoriasis compared with controls (9–20). Studies that defined enthesitis by the Glasgow Ultrasound Enthesitis Scoring System (GUESS) and Outcome Measures for Arthritis Clinical Trials (OMERACT) reported significantly higher scores in patients with psoriasis (12, 14, 16). Subclinical enthesopathy was detected in five studies (9, 12, 13, 15, 18, 19), which highlights a potential role for US in screening asymptomatic patients with psoriasis for musculoskeletal abnormalities.

Ten out of the 13 cross-sectional studies assessed the Achilles tendon by US, making it the most common site being evaluated (9, 10, 12–16, 18–20). Eight of these studies demonstrated a higher proportion of patients with Achilles tendinitis and enthesopathy in the psoriasis group compared with controls (9, 10, 12–16, 18). In addition to Achilles enthesitis, one study also found a higher proportion of sonographic enthesopathy at the proximal patellar tendon (*p* < 0.0005), distal patella tendon (*p* = 0.008) and plantar fascia in the psoriatic group (13). Bursitis was cited as the second most common sonographic abnormality by two studies (11, 16).

Doppler signal was also significantly higher in psoriatic patients with US findings of Achilles enthesopathy (12, 13). Only one study focused on contrast enhanced US, which showed a good concordance with MRI findings and helped increased diagnostic confidence (21).

Interestingly, nail involvement was found to correlate with sonographic abnormalities in three studies, more specifically with enthesopathy at the distal interphalangeal joint (17), higher OMERACT enthesopathy scores (14), subclinical enthesopathy (19) and active sonographic synovitis and enthesitis (20).

Three studies followed patients with psoriasis who had undergone a baseline US longitudinally to identify features predictive of progression to PsA. One study followed up psoriatic patients with or without symptoms of arthralgia at 3 months (20). The incidence of progression to PsA was 109.2/1,000 person-years for psoriatic patients who had arthralgia, vs. 13.4/1,000 person-years for those without (*p* = 0.03). The presence of sonographic enthesitis at baseline correlated with progression to PsA (*p* = 0.03). Another study with follow-up of 2 years found annual incidence of PsA to be 4.3% in the psoriasis group. Psoriatic patients who were more likely to develop PsA had a higher prevalence of baseline enthesitis, higher C reactive protein levels, and higher PD and gray-scale synovitis scores (22).

In the third study 7/28 (23%) patients fulfilled a diagnosis of PsA at follow-up at 3.5 years, with a median of 13 months between baseline US and development of PsA. Consistent with the other studies baseline enthesitis scores were significantly higher in those who developed PsA. Logistic regression analysis found that baseline quadriceps tendon thickness was an independent predictor of PsA development (*p* = 0.029) in this cohort (23).

## Differential Diagnosis: Ultrasound Findings in Psoriatic Arthritis in Comparison With Other Conditions

US could be a useful tool in differentiating PsA from other conditions such fibromyalgia, psoriasis and other inflammatory arthritides. In total, 24 studies were identified in this review that evaluated US abnormalities in PsA and compared them to other conditions. i.e., psoriasis (10 studies), RA (7 studies), fibromyalgia (3 studies), other spondylarthritis (SpA) and OA (2 studies each). A summary of the US findings that differentiate PsA from other conditions as reported in the studies included in this review is depicted in Table 1.

**Table 1:** Summary of ultrasound findings that differentiate psoriatic arthritis from other conditions.

	**Ultrasound (US) findings that differentiate psoriatic arthritis (PsA) from other conditions**
Psoriasis	• US inflammatory and chronic changes in joints, tendons and entheses are more frequent in PsA compared to. • Most specific US features of PsA are Power Doppler signal and erosions in joints and entheses. • Achilles tendon insertion is the entheseal site most frequently involved in PsA compared to psoriasis. • Nail US inflammatory and structural abnormalities are more frequent in PsA and psoriasis compared to health controls, but there are no significant differences between the two groups.
Rheumatoid arthritis	• Extrasynovial changes (capsular enthesophyte, juxta-articular periosteal reaction, enthesopathy at the site of deep flexor tendon insertion on the distal phalanx, and subcutaneous soft tissue thickening of the finger pad or entire finger) are specific for PsA. • Tenosynovitis, peri-tendinous dermal soft tissue oedema with associated Power Doppler signal and flexor tendon enthesopathy including new bone formation at the insertional site are specific for PsA. • Thickening of the pulleys in the flexor tendons seems to be a characteristic feature of PsA and has a good sensitivity for the diagnosis of PsA.
Fibromyalgia	• US-detected entheseal abnormalities in particular erosions and Power Doppler signal are more frequent in patients with PsA compared to fibromyalgia. • Involvement of the Achille's tendon, in particular inflammation, seems to be specific for PsA.
Other spondylarthritides	• Power Doppler enthesitis is a frequent finding in active axial spondyloarthritis and PsA, but there is no significant difference between the two conditions. • The presence of bursitis in the 5th metatarsophalangeal joint might be a distinctive clinical sign of PsA.
Osteoarthritis	• The frequency of calcaneal enthesophytosis is similar in osteoarthritis and PsA, but inflammatory lesions of calcaneal entheses and of the adjacent bursae are more frequent in PsA. • Thickening of pulleys in flexor tendons is more frequent in PsA than osteoarthritis.

### Psoriatic Arthritis vs. Psoriasis

Ten studies evaluating the differences of US findings in PsA vs. psoriasis were identified in this review (15, 24–32). Enthesis was the most frequently evaluated anatomical structure by US (6 out of 10 studies).

Most studies showed that US changes, both inflammatory and structural, are more frequent in patients with PsA compared with psoriasis only (15, 24, 25, 27, 30, 32). However, as stated above, subclinical US inflammatory changes are quite common in patients with psoriasis without PsA (9–16, 18, 20). The most important specific US features of PsA were PD signal and erosions in joints and entheses (15, 24, 25, 32).

Some of the studies reported some differences in the entheseal sites most affected by PsA compared to psoriasis: Achilles tendon insertion (27, 32), followed by the tibial tuberosity insertion and plantar aponeurosis insertion (27). In another study, the most discriminative site for PD positivity in PsA was the retrocalcaneal bursa (15).

Three studies evaluated nail involvement in PsA and psoriasis (26, 28, 29), all reporting a higher frequency of US inflammatory and structural abnormalities, in both groups compared to healthy controls, but no significant differences were found between PsA and psoriasis.

### Psoriatic Arthritis vs. Rheumatoid Arthritis

Seven studies that compared US features in PsA to RA patients were included in this review (33–35). Several US findings that clearly distinguish between the two conditions were identified.

While synovitis and tenosynovitis were seen in both PsA and RA, extra-synovial changes were specific for PsA (36–38). The extra-synovial changes include findings such as capsular enthesophyte, juxta-articular periosteal reaction, enthesopathy at the site of deep flexor tendon insertion on the distal phalanx, and subcutaneous soft tissue thickening of the finger pad or entire finger. In a study comparing the US appearance of the hand flexor tendon compartment in PsA to RA patients (39), the following findings were identified as being specific for PsA: peri-tendinous dermal soft tissue oedema with associated PD signal (30% in PsA vs. none in RA), flexor tendon enthesopathy including new bone formation at the insertional site (65% in PsA vs. 9% in RA), and tenosynovitis (38% in PsA vs. 13% in RA). Similarly, in other studies, extra-synovial changes were found in 84% fingers with PsA vs. none of the fingers with RA (36) and peritenon extensor tendon inflammation was identified in 65.8% metacarpophalangeal (MCP) joints in PsA vs. none in RA (37). Likewise, Zabotti et al. (35) have shown that patients with early PsA displayed a more common US extrasynovial and synovio-entheseal complex involvement compared to early RA. The detection of at least one extra-synovial US feature (i.e., peritendon inflammation of the extensor digitorum tendon at MCP joint, central slip enthesitis at the proximal interphalangeal joint, and soft tissue oedema around flexor tendon of the digit showed good sensitivity (68%) and a specificity (88.1%) for early PsA vs. early RA.

Involvement of pulley in the flexor tendons was also more frequently seen in patients with PsA: the thickening of the pulleys had good sensitivity (80%) for the diagnosis of PsA (38); in another study, inflammation of A1 pulley was present in 15 of 240 fingers (6.3%) of eight PsA patients (26.7%) and in one of 240 fingers (0.4%) of one RA patient (3.3%), suggesting that it might be a characteristic feature of PsA compared to RA (34).

### Psoriatic Arthritis vs. Fibromyalgia

Differentiating enthesitis due to PsA from widespread pain due to fibromyalgia remains a challenge in clinical practice; moreover, the two conditions frequently overlap. In this review we have identified three studies that have evaluated the role of US in distinguishing between PsA and fibromyalgia (40–42).

All studies showed that US-detected entheseal abnormalities are more frequent in patients with PsA (ranging from 70 to 90% of the patients) than in fibromyalgia (23–35.3%) (40–42). The most specific US findings for PsA were entheseal erosions and PD signal, which were present only in patients with PsA and in none of the patients with fibromyalgia (40, 41).

The entheseal sites most specific for PsA in comparison to fibromyalgia was the insertion of the Achilles tendon (40–42), followed by quadriceps and patellar tendons (40, 42) and the plantar fascia (41).

### Psoriatic Arthritis vs. Other Spondyloarthritides

Only two studies (43, 44) assessed the role of US in distinguishing PsA from other forms of SpA. Molina Collada et al. showed that while PD enthesitis was a frequent finding in active axial SpA and PsA, there was no significant difference between the two conditions (43). However, the presence of bursitis in the fifth metatarsophalangeal joint might be a distinctive clinical sign of PsA, useful for differential diagnosis with the other SpA. In a cross-sectional study including 150 patients with PsA, 172 with SpA and 95 healthy controls, bursitis was diagnosed in 11.3% PsA patients but in none of the SpA and healthy controls (44).

### Psoriatic Arthritis vs. Osteoarthritis

Although differentiating PsA from OA can be challenging in clinical practice, there were only two studies identified in this review to compare US findings in these two conditions. Falsetti et al. have assessed the prevalence of calcaneal entheses involvement in erosive OA, nodal OA, RA and PsA using US (33). The frequency of calcaneal enthesophytosis was similar in erosive OA, nodal OA and PsA, but inflammatory lesions of calcaneal entheses and of the adjacent bursae, i.e., hypoechogenicity, thickening of the Achilles tendon enthesis and deep retrocalcanear bursitis were more frequent in RA and in PsA. Furlan et al. have assessed the A1 pulleys of digital flexor tendons in 206 patients with various conditions (38). Out of the 86 patients that presented thickening of pulleys, 46.5% had PsA and 14% had OA. This US finding had a good sensitivity for the diagnosis of PsA, suggesting that it might be used in clinical practice to differentiate PsA from OA and other conditions. However, generalization of these results must be made cautiously, as confounders such as diabetes or manual work haven't been evaluated in these studies.

## Discussion

The role of US in the early diagnosis of PsA is still unclear, despite growing evidence of increased sensitivity compared to clinical examination. Subclinical synovitis and enthesitis is common in patients with psoriasis. This literature review highlights the ability of US to detect subclinical synovial and entheseal inflammation in patients with psoriasis. The existing literature showed a higher frequency of sonographic abnormalities in patients with psoriasis compared with controls, with the area most commonly interrogated by US being the Achilles tendon enthesis. Absence of doppler signal at entheseal sites for control patients in all the studies of this review supports the use of PD in combination with gray-scale US that captures structural damage. This is consistent with literature demonstrating that the addition of PD increases diagnostic accuracy for (45, 46). The Group for Research and Assessment of Psoriasis and Psoriatic Arthritis (GRAPPA) Ultrasound Working Group is developing a preliminary ultrasonographic enthesitis score in PsA, which incorporates Doppler signal in addition to structural entheseal changes (47).

Interestingly, multiple studies in this review highlighted that sonographic enthesopathy was associated with psoriatic nail involvement. Given the close relationship between the nail and the enthesis of the distal interphalangeal extensor tendon (48), GRAPPA has proposed an ultrasonographic index for nail enthesis assessment as well (49). However, this instrument has not been validated by any other group and includes the problematic measurement of skin over the cuticle in the measurement of the nail matrix.

Though there were limited longitudinal studies, it is evident that patients with psoriasis most likely to develop PsA had higher levels of enthesitis, pain scores and sonographic abnormalities at baseline. Further studies would help strengthen the evidence supporting the use of US as a screening tool in patients with psoriasis to facilitate early detection and management of PsA.

This review has also identified specific US features that could help differentiating patients with PsA from various other conditions. Patients with PsA have a higher prevalence of US findings, both inflammatory and structural in all examined structures in comparison with patients with psoriasis only, OA, and fibromyalgia.

Most specific US features of PsA were PD signal and erosions in joints and entheses. Achilles tendon insertion was the site most frequently involved in PsA. These findings are of importance especially in the enthesitic predominance subset of PsA, where there is less clinical evidence of inflammation. Enthesitis is a hallmark feature of PsA, however, it is challenging to ascertain in clinical practice whether clinical enthesitis is due to soft-tissue inflammation, structural damage, presence of pain dysregulation or fibromyalgia (50, 51). The biomechanical factor related to obesity is an important confounder in these studies, as entheseal imaging cannot discriminate between PsA, psoriasis and healthy subjects with high body mass index (52).

The presence of concurrent fibromyalgia has been shown to be linked with higher clinical enthesitis scores, without an increase in US inflammation, suggesting that imaging, including US, should be the preferred modality to detect enthesitis in PsA patients with concurrent fibromyalgia rather than clinical examination (53). However, some confounding factors such as gender or body mass index should be considered in these studies too, given the different characteristics between patients with fibromyalgia vs. psoriasis and/ or PsA.

PsA patients share some similar US features with RA patients, such as synovitis, tenosynovitis, and erosions, however there are features specific for PsA (enthesitis, peritendonitis of extensor tendons of hands, pulley thickening, peri-tendinal dermal oedem) in keeping with the physio-pathological and clinical concept of “synovio-entheseal complex” which is characteristic for PsA (54, 55). Applying this finding in clinical practice could contribute to an early and accurate diagnosis and subsequently to a better management of patients with PsA. There is less data on using US in the differential diagnosis between PsA and other SpA and OA, respectively. This remains an important unmet need as it is frequently encountered in clinical practice.

### Limitations, Unmet Needs, and Research Agenda

These results are of great clinical importance with respect to PsA early and differential diagnosis. However, the majority of patients included in these studies had established PsA or there was no distinction between early and established PsA. Further research is warranted to determine whether the specific US features that distinguish PsA from other conditions are characteristics for early PsA as well.

A limitation of the studies included in this review is the high heterogeneity of the studies in terms of included population, US elementary lesions and sites examined, and US scores. While most studies included patients with PsA according to CASPAR criteria, some used other criteria such as Moll and Wright criteria or the rheumatologist's opinion. There was a high variability of structures and sites examined, e.g., some studies evaluated just entheses or only one joint/ area of interest such as the knee or the distal interphalangeal joints in the hands. Lastly, only some of the studies used global enthesitis US scores. There is no consensus on what US scores to use in PsA as they vary considerably with regards to sites included and elementary lesions evaluated, e.g., not all scores capture or distinguish between active inflammatory and chronic damage changes. Hence, the generalizability of these results should be regarded cautiously, and further efforts should be made for consensus on a standardized US evaluation of patients with PsA. GRAPPA is currently working on a developing a sonographic enthesitis scoring system that would reliably identify PsA at early stages, thus optimizing early diagnosis and encouraging timely interventions (56). Moreover, the studies span many years and US technique has improved over this time. This heterogeneity explains well the large discrepancies in the results of these studies and makes it difficult to compare across studies.

None of the studies included in this review have evaluated the added value of US to clinical and laboratory information in early identification of PsA, differential diagnosis, or disease assessment. Addressing this research gap is important to determine the role of US within the routine practice.

**Figure 1:**
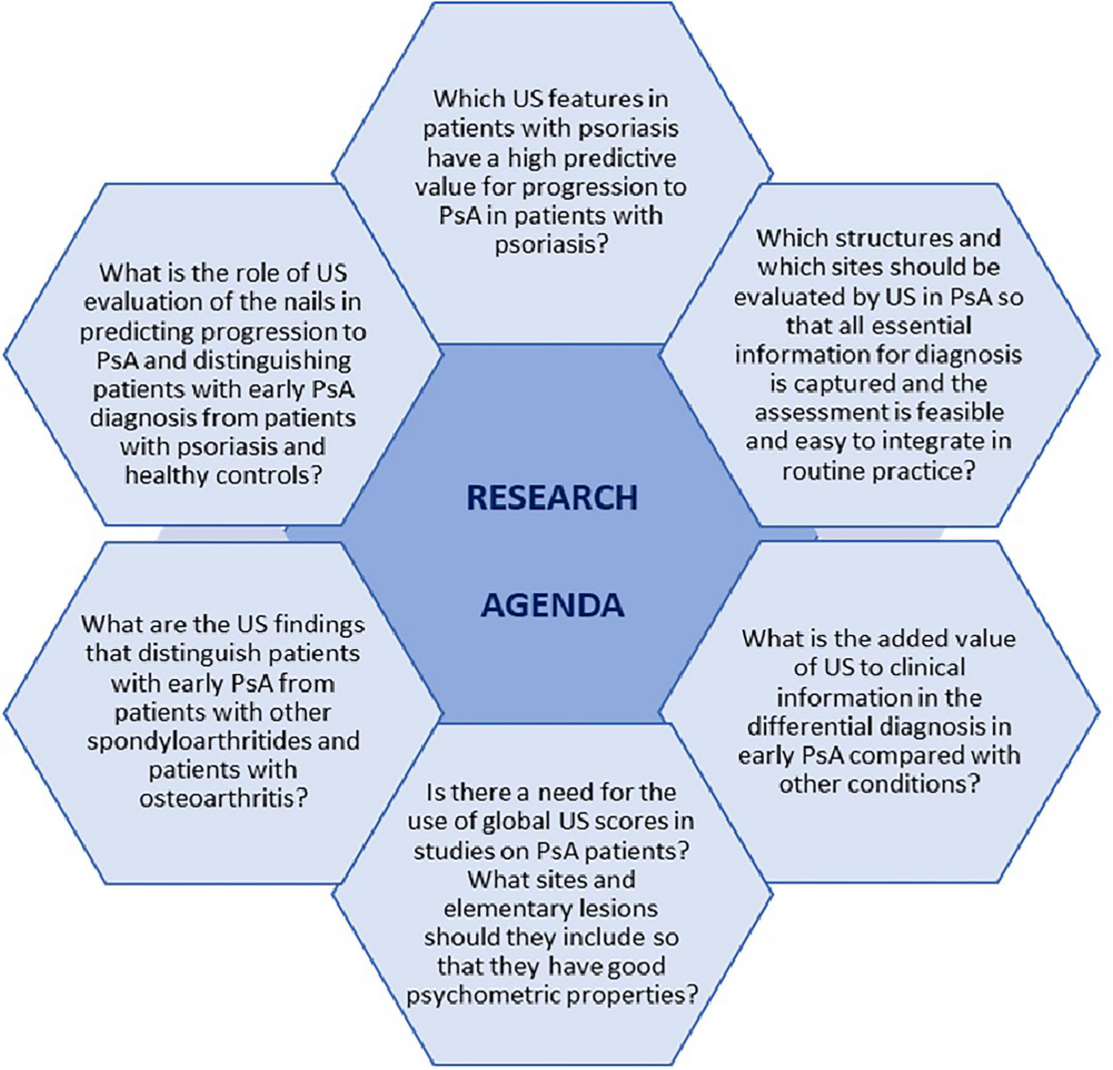
Research agenda for the use of ultrasound in early psoriatic arthritis.

This review illustrates the importance US in early PsA. The conclusions of the included studies demonstrate that US is a valuable tool in identifying patients with psoriasis who will further develop PsA, as well as in differentiating PsA from other conditions. However, further research is needed to improve and optimize the use of US in the early diagnosis of PsA. Main unmet needs and questions on research agenda are depicted in Figure 1. Efforts should be made in designing more longitudinal studies to assess which US features have a high predictive value for progression to PsA in patients with psoriasis. Moreover, identifying which anatomical structures, elementary lesions, and sites are specific for PsA, as well as developing validated sonographic scores are essential for early diagnosis of PsA and for setting up standardized, consistent US-based studies.

## Conclusion

US is a useful instrumental for the diagnosis of early stages of PsA, thus contributing to a better management of the disease and to better patient outcomes. However, there are several limitations and unmet needs that need further research and improvement.

## Author Contributions

TG, BN, HJ, MS, and DJ contributed to methodology and to the writing of the manuscript. TG, BN, HJ, CG, VK, JR, and CZ have performed the literature review and data extraction. All authors discussed the results and commented on the manuscript.

## Conflict of Interest

The authors declare that the research was conducted in the absence of any commercial or financial relationships that could be construed as a potential conflict of interest.

## Publisher's Note

All claims expressed in this article are solely those of the authors and do not necessarily represent those of their affiliated organizations, or those of the publisher, the editors and the reviewers. Any product that may be evaluated in this article, or claim that may be made by its manufacturer, is not guaranteed or endorsed by the publisher.
