# Supplementary material for: Improving Imaging Modalities in Early Psoriatic Arthritis: The Role of Ultrasound in Early Diagnosis of Psoriatic Arthritis
Source: Front Med (Lausanne). 2022 Jan 7;8:804695. doi: 10.3389/fmed.2021.804695 (PMC8776996; doi:10.3389/fmed.2021.804695)
Supplement: Supplementary file 1 [file Table_1.docx]

**Supplementary table 1:** Characteristics of studies on ultrasound in patients with psoriasis

| **Author, Year, Country** | **Study type** | **Study population** | **Site(s) examined by ultrasound** | **Doppler** | **Findings** |
| --- | --- | --- | --- | --- | --- |
| De simone et al, 2002  Italy | Cross-sectional | Psoriasis (n=59, 15 have PsA)  Control (n=59) | Achilles tendon (enthesis) | No | Ultrasound found Achilles tendinitis in a higher proportion of psoriatic patients (59.3%) compared with clinical evaluation alone (30.5%). 53.6% asymptomatic patients demonstrated sonographic Achilles tendonitis, in addition to retrocalcaneal bursitis. |
| Özçakar et al, 2005  Turkey | Cross-sectional | Psoriasis (n= 30, 11 have PsA)  Control (n= 20) | Achilles tendon (enthesis) | No | Achilles tendon mean thickness was significantly higher in patients with psoriasis versus controls (4.44±0.64 vs 4.09±0.26, p<0.05) and in patients symptomatic of enthesitis (p=0.001). |
| De simone et al, 2011  Italy | Cross-sectional | Psoriasis with finger and/or toe pain (n=52)  Psoriasis without musculoskeletal pain (n=50) | MCP, PIP, DIP of fingers, MTPs, PIP and DIP of toes  Flexor and extensor tendons of fingers and toes | Yes | A total of 36 patients were diagnosed with PsA amongst the 52 patients with painful fingers and/or toes, of which all had abnormal sonographic features and 29 of the 36 patients had increased power doppler signal, particularly at the flexor tendons of affected digits. Only 11 of the 36 patients had radiographic features of PsA. None of the asymptomatic patients had sonographic findings suggestive of PsA. |
| Gutierrez et al, 2011  Italy | Cross-sectional | Psoriasis (n=45)  Control (n=45) | 5 entheses bilaterally:  Achilles tendon, quadriceps insertion into upper pole of patella, patellar tendon insertion into lower pole of patella, plantar aponeurosis | Yes | Asymptomatic psoriatic patients had significantly higher GUESS scores (p<0.0001) and doppler signal at entheses sites (p<0.0001) compared with controls. Doppler signal was absent in control patients. Achilles enthesis was the site most common for enthesopathy. |
| Naredo et al, 2011  Spain | Cross-sectional | Psoriasis (n=162)  Control (n=60) | 36 joints: wrist joints, MCP joints, PIP and DIP joints of the hands, knee and tibiotalar joint  22 tendons: wrist extensor compartment, finger flexor tendons  18 entheses: Achilles tendon, proximal and distal patellar tendon, plantar fascia, deep flexor tendons of fingers | Yes | There was significantly more frequent sonographic synovitis (p=0.024), enthesopathy (p=0.005), entheseal power doppler signal (p=0.013) in psoriatic patients compared with control. 3.2% psoriasis patients and 1.3% in control group had sonographic synovitis (p<0.0005), 11.6% psoriasis patients and 5.3% in the control group had sonographic enthesopathy. Entheseal power doppler signal was only present in psoriatic patients. |
| Ash et al, 2012  United Kingdom, Italy | Cross-sectional | Psoriasis with nail involvement (n=31)  Psoriasis without nail involvement (n=15) Control (n=21) | Entheses: upper limb (common extensor tendon origins) and lower limbs (Achilles, plantar fascia insertion, quadriceps insertion, patellar tendon origins and insertions) | Yes | Higher enthesitis scores were present in psoriasis patients compared with controls (21 vs 11, p=0.005). Enthesopathy scores (OMERACT) were higher in patients with nail disease (p=0.02). The duration of psoriasis correlated with enthesitis (p=0.05). Higher mNAPSI scores correlated with sonographic features of inflammation and chronicity. |
| Aydin et al, 2012  United Kingdom, Italy | Cross-sectional | Psoriasis (n=42)  PsA (n=58) Control (n=23) | Lower limb entheses: Achilles, plantar fascia, quadriceps, patellar tendon origins and insertions | Yes | Psoriatic patients had higher enthesitis scores than controls (p<0.0001). PsA patients had higher sonographic scores than psoriasis patients. Doppler positivity was more frequent in the PsA group vs the psoriasis group (36.2% vs 9.5%, p=0.002). Positive Doppler has a specificity of 91% and positive likelihood ratio of 3.8 to discriminate PsA and psoriasis. Subclinical enthesopathy was detected at higher rates in PsA than psoriasis alone. |
| Pistone et al, 2014  Italy | Cross-sectional | Moderate to severe plaque psoriasis (n=59)  Control (n=59) | Achilles tendon (enthesis) | No | Patients with psoriasis had significantly higher GUESS scores, with 22% demonstrating thickened Achilles tendon (>5.29mm) and irregular tendon structure. Bursitis was the second most commonly found abnormality, with 12% of psoriatic patients affected in this study. |
| Castellanos-González et al, 2016  Spain | Cross-sectional | Psoriasis with nail involvement (n=61)  Psoriasis without nail involvement (n=29) | DIP joints of hands | Yes | Enthesopathy was present in 82.6% of patients with nail involvement and 17.4% in those without. tNAPSI score ≥4 had a sensitivity of 60.9% and specificity 71.6% for predicting sonographic DIP enthesopathy. |
| Oğuz et al, 2016  Turkey | Cross-sectional | Psoriasis (n=50)  Control (n=30) | Joints: bilateral shoulders, elbows, knees  Tendons: flexor and extensor tendons of all fingers  Entheses: Achilles tendon and plantar fascia | Yes | There was a higher prevalence of pathological sonographic findings in the psoriasis group compared with controls (30% vs 13.33%, p>0.05). Though not statistically significant, this study shows abnormalities in asymptomatic patients. |
| Moya Alvarado et al 2018  Spain | Cross-sectional | Psoriasis (n=48) | Nails of dominant hand  Entheses: Achilles tendon, extensor digitorum | Yes | 68.8% patients had sonographic evidence of extensor digitorum tendon enthesopathy. Patients with subclinical enthesopathy had greater nail involvement, with thicker proximal nail folds (p=0.023). |
| Zabotti et al, 2019  Italy | Cross-sectional | Psoriasis (n=57)  Psoriasis with arthralgia (n=61)  Controls (n=57) | Joints: MCP, PIP, DIP of hands, wrists, knees, MTP joints  Entheses: Achilles, quadriceps, proximal and distal patellar, plantar aponeurosis and common extensor tendon  Bursae: retro-calcaneal  Tendons: extensor and flexor digitorum tendons of hands, extensor tendon compartment of wrist | Yes | In psoriatic patients with arthralgia, 14.7% demonstrated sonographic entheseal erosions, compared with 5.3% of psoriatic patients without arthralgia (p=0.13) and none of the control group (p=0.003). In psoriatic patients with arthralgia, patients with active sonographic synovitis and enthesitis had significantly higher NAPSI scores.  Higher sonographic enthesopathy scores were also associated with non-smokers and patients with a higher pain VAS. |
| Solivetti et al, 2010  Italy | Cross-sectional | Patients with psoriasis who have suspected PsA n=22  Control n=10 | Joints suspicious for PsA | Yes | Contrast enhanced ultrasound when utilised, increased diagnostic confidence in symptomatic cases with negative diagnosis. 90.9% of suspected PsA cases had positive contrast enhancement. In addition there was 100% concordance between contrast enhanced ultrasound and MRI findings. |
| Tinazzi et al, 2011  Italy | Longitudinal (3.5 year follow-up) | Psoriasis (n=28) | Entheses: quadriceps tendon, proximal patellar ligament, distal patellar ligament, Achilles tendon, plantar aponeurosis | Yes | 7 of 28 patients (23%) fulfilled CASPAR criteria for the diagnosis of PsA at follow-up, with a median of 13 months between baseline ultrasound and development of PsA. Baseline GUESS scores of patients who developed PsA were significantly higher. Logistic regression |
| Elnady et al, 2019  Saudi Arabia | Longitudinal (2 year follow-up) | Psoriasis without clinical signs of PsA (n=109)  Controls (n=90) | 8 entheses, 34 joints: bilateral wrists,  metacarpophalangeals MCP, PIP, DIP, thumb IPJ, knees, and ankles | Yes | Annual PsA incidence was 4.3%. Psoriatic patients who were more likely to develop PsA had higher prevalence of baseline enthesitis, higher CRP levels, higher power doppler and gray-scale synovitis scores. |
| Zabotti et al, 2019  Italy | Longitudinal (3 months) | Psoriasis (n=48)  Psoriasis with arthralgia (n=54) | Joints: MCP, PIP, DIP of hands, wrists, knees, MTP joints  Entheses: Achilles, quadriceps, proximal and distal patellar, plantar aponeurosis and common extensor tendoncrp  Bursae: retro-calcaneal  Tendons: extensor and flexor digitorum tendons of hands, extensor tendon compartment of wrist | Yes | Development of PsA occurred in 5/54 patients who had psoriasis with arthralgia at baseline, compared with 1/48 patients with psoriasis alone. The incidence rate is 109.2/1000 person years in psoriasis patients with arthralgia and 13.4/1000 person years in psoriasis (p=0.03). Patients developing PsA had significantly higher baseline VAS, HAQ and joint tenderness. Sonographic enthesitis was associated with progression to PsA (p=0.03). |
| Legend:  PsA = psoriatic arthritis, OMERACT = Outcome Measures for Arthritis Clinical Trials, GUESS = Glasgow Ultrasound Enthesitis Scoring System, MCP = metacarpophalangeal, PIP = proximal interphalangeal, DIP = distal interphalangeal, IPJ = interphalangeal joint, tNAPSI = target nail psoriasis index, mNAPSI = modified nail psoriasis index, MTP = metatarsophalangeal, VAS = visual analogue score, CASPAR = Classification of Psoriatic Arthritis, CRP = C reactive protein, HAQ = health assessment questionnaire | | | | | |
